# Deconstructing the masculinized assumption of the medical profession: narratives of Japanese physician fathers

**DOI:** 10.1186/s12909-023-04855-4

**Published:** 2023-11-12

**Authors:** Noriki Kamihiro, Futoshi Taga, Junichiro Miyachi, Tomoko Matsui, Hiroshi Nishigori

**Affiliations:** 1 Center for Medical Education, Graduate School of Medicine, Nagoya University, 65 Tsurumai-cho Showa-ku Nagoya, Aichi, 466-8560 Japan; 2 Department of Education and Culture, Faculty of Letters, Kansai University, Osaka, Japan; 3 Academic and Research Centre, The Hokkaido Centre for Family Medicine, Hokkaido, Japan; 4 Department of Family and Community Medicine, Hamamatsu University School of Medicine, Shizuoka, Japan

**Keywords:** Gender issues in the medical profession, Masculinity, Gendered assumptions, Professionalism, Family life of doctors, Patriarchy

## Abstract

**Background:**

Gender studies in the medical profession have revealed gender biases associated with being a doctor, a profession often regarded as more suitable for men. The path to gender equality inevitably involves deconstructing this masculinized assumption. Despite the decades-long expectation that *ikumen*–men who actively participate in childcare in Japan–would contribute to a change toward gender equality, Japanese society is still male dominated, and women suffer from a large gender gap. With the aim of exploring implicit gendered assumptions concerning being a caregiver and a doctor, the authors focused on the experience of individuals juggling the binary roles of a professional and a caregiver.

**Methods:**

The authors conducted subjectivist inductive research, recruited ten Japanese physician fathers through purposive sampling, and collected data through one-to-one semi-structured interviews between October 2017 and December 2018. The authors recorded and transcribed the narrative data, and extracted themes and representative narratives.

**Results:**

The study identified three themes about the reproduction and potential change of the gender gap: maintaining gendered assumptions of the medical profession without experiencing conflict, maintaining gendered assumptions of the medical profession while experiencing conflict, and deconstructing gendered assumptions of the medical profession through conflict. The authors found that these negotiations interplayed with the gendered division of labor between male doctors and their wives as well as the patriarchal family structure.

**Conclusions:**

The study revealed how gendered assumptions of the medical profession, as well as gender stereotypes and gendered division of household labor, were reproduced in the course of male doctors’ negotiations when they became fathers. For male doctors to question their unconscious gender bias, the authors emphasize the importance of men gaining knowledge about gender stereotypes, and propose that educators create such opportunities. Moreover, the authors assert that increasing doctors’ awareness of how masculinized assumptions implicitly interact with ideas of being a doctor—an aspect rarely discussed among medical professionals—is crucial for deconstructing the gendered normativity in the medical field.

## Background

Gender equality in the medical profession is of increasing concern for female doctors facing gender-related difficulties. Previous research has highlighted their experiences of gender inequality and segregation in professional communities. Female doctors: are paid less than male doctors [1]; suffer unequal treatment due to gender stereotypes, such as “physicians should be men” and “women must prioritize family responsibilities” [2]; and report witnessing and experiencing sexism at work [3]. While other professions can entail gender-based inequalities [4, 5], the medical profession is particularly unique in that gender influences the professionals’ processes of forming professional identities. Some scholars have pointed out that professional identity formation, considered a major factor throughout the continuum of medical education [6], is influenced by specific gender norms [7–11]. For example, female medical students and physicians struggle to reconcile their professional and gender identities in a masculinized professional community [8] and in a society that, largely, has a patriarchal family structure (10). Women have more difficulty finding mentors [7], which is crucial for leaners to reflect on experiences and develop professional identity [9].

These preceding studies have yielded answers to the question of *whose* standards doctors conform to; men’s [7, 12–14]. In contrast, research on *how* the link between masculinity and the medical profession is maintained is scarce, and so is insight into *how* this link can be dismantled. Ozbilgin et al. researched this unexplored area, and investigated the connectivity of gender and time in norms regarding professional behavior to reveal how a certain discourse of professionalism was used to retain and reproduce gender order at work [15]. As they suggested in their article [15], the next step in this area should be to focus on the discourses of *standard* work ethics in health professions and examine the assumptions underpinning them.

In addition to these examinations into gender structure within the medical profession, clues to further understanding the gender gap among doctors may be found in the interactions between their professional and domestic roles. In their review, where they presented several theories and frameworks to explain the division of household labor [16], Shelton and John argued that individuals with more resources (such as education, income, and occupational prestige), and those with more time constraints owing to work, are more likely to be exempted from household chores. Accordingly, physicians, typically having such resources [17, 18] and working for long hours [19], are more likely to be excused from domestic duties.

Considering this interrelationship between doctors’ work and family roles, we examine how this relationship evolves when physicians assume an increased amount of household labor. Particularly, to explore this more comprehensively, we examine male doctors involved in childcare in Japan. Since the mid-20th century, Japanese families have followed a gendered division of labor, where men have been less involved in caregiving and household chores compared to women. While the number of dual-income households was nearly three times higher than that of single-income households with stay-at-home wives as of the year 2022, the situation remains largely unchanged, with men dedicating minimal time to caregiving or household responsibilities [20, 21]. These disparities in domestic roles between husbands and wives have been reported to be similar for physician couples [22, 23]. Even in 2023, Japan ranks at among the lowest levels on the Global Gender Gap Index (125th of 146 countries) [24]. Nevertheless, amid this social background, there has been a new trend regarding fathers’ participation in childcare since the 2000s. Fathers who actively took on the responsibility of caring for their children became applauded, accompanied by the new term “*ikumen*” to denote such men. In recent masculinity studies, this form of masculinity, which adopts care-related characteristics traditionally considered feminine (i.e., caregiving, domestication, etc.), is referred to as a caring masculinity [25, 26]. With the widespread adoption of this trend across Japan, the Japanese medical community also saw an increase in the number of physician fathers adopting this role, resulting in the emergence of a master narrative on childcare by male doctors, often described as a positive and welcoming attitude [27, 28]. However, as outlined earlier, this new form of masculinity symbolizing hope for gender equality has not substantially influenced the gender structure at the macrosocial level. Why, then, has it been challenging to effect societal level changes when men have been increasingly making new adjustments to their gendered roles? How can this challenge be overcome?

Based on these arguments, regarding the professional standards that physicians are expected to meet, we presumed that there would be implicit gendered assumptions which may inhibit the fight at the individual level toward changing gender norms, thus sustaining the gender gap. With the aim of exploring how gendered assumptions interplay with individuals’ negotiations, our study specifically examined the experience of Japanese physician fathers struggling between the binary roles of working, which has been attributed to men, and childcare, which has been considered feminine. Therefore, we designed a qualitative study with the research question, “When male doctors become fathers, how do their negotiations about being a caregiver interact with gendered assumptions about being a doctor?”

## Methods

### Study design and theoretical orientation

Since this study aimed to explain a particular phenomenon and develop new insights by focusing on individual experience, we adopted a subjectivist inductive approach, which begins not with a hypothesis but with collecting data about the phenomenon and search for patterns across the data to generate an understanding of the phenomenon [29]. However, we allowed, in the course of research, the adoption of particular theories or concepts to inform the research process, if the researchers found that those theories or concepts could shape the research better [29].

Based on this position, we employed the following concepts, which we acknowledged as appropriate for the proposed research question, enhancing the coherence, logic, and relevance of this study vis-à-vis previous research in this academic area: Gender is constructed through interactions between individuals and society in a specific social context [30]. The male gender is not uniform, yet there is a plurality of masculinities and a hierarchy within these masculinities [31]. Patriarchy refers to a form of organizing a society with power relations between male dominance and female subordination [32, 33]. The gendered division of labor refers to the allocation of different types of work or tasks between women and men; we used this word in our article as the allocation of roles between participants and their wives, which assumed that men should work outside the home and women should do household work [10, 22, 32]. In addition to these concepts, we also define childcare. Drawing on previous research [20, 21, 25, 34, 35], we define childcare as a concept encompassing three types of activities, including caregiving that involves direct physical contact; domestic work associated with nurturing children, such as cooking and doing laundry; and interacting with children in forms, such as playing. Each of these activities requires caregivers to assume varying levels of responsibility and perform them at different levels of frequency. Some high responsibility tasks must be performed on a daily basis, while other tasks are less demanding and can be performed at the caregivers’ own pace.

### Research participants

We adopted a purposive sampling method to recruit the participants. We contacted the candidates via email through referrals from the researchers’ friends, acquaintances, and colleagues. All the candidates worked full- or part-time at clinics or hospitals in Japan. Those who agreed to participate in this study and met all three of the following criteria were included: Japanese male physicians who were (1) in a heterosexual marriage, (2) engaged in childcare for at least one hour daily, and (3) residing with their child under the age of seven. The first criterion was set because we deemed it necessary to distinguish between the childcare experiences of men in male-female couples and those of single parents or same-sex couples; our study focused on the former. It is important to note that Japan has a relatively low rate of births occurring outside of marriage (2–3%), in contrast to the average of OECD countries (42%) [36]. Additionally, Japanese law does not recognize same-sex marriage, limiting adoption options for same-sex couples [37]. The second criterion was set based on the median domestic work hours (including childcare, family care, and chores) of male doctors in Japan, which is three hours per week [22], to select those who were more engaged in childcare than average men. The third criterion was informed by the observation that in Japan, the amount of time spent on childcare tends to decrease significantly after the age of six [20]. We did not include the wives’ employment status as a selection/exclusion criterion, as our intention was to explore the general experiences of male physicians in Japan. Hence, most participants had wives who were either full-time housewives or part-time workers. It is important to acknowledge that this group of participants may not represent the reality of male physicians whose wives are full-time employees or those who are single parents raising children on their own. Nevertheless, given the statistical trend that most Japanese male physicians’ spouses are housewives [23], this participant group served to capture the general experiences of male physicians in Japan.

### Data collection

Semi-structured interviews were conducted in Japanese, either face-to-face or online between October 2017 and December 2018. Each interviewee participated in a single interview session. The duration of each interview ranged from 39 to 68 min. All interviews were conducted one-on-one by NK, except for that of Akira (a pseudonym), who was interviewed by two researchers (NK and TM). The interviewer explored the experiences of the participants using an interview guide prepared in advance (Table 1), and asked additional questions when relevant or when important narratives emerged. All interviews were recorded on a digital audio recorder. Verbatim transcripts were created from the audio-recorded data.

**Table 1:** The interview guide used during the semi-structured interviews

1. Family members
‘Can you tell me about your family members?’
2. Roles and contributions in the family
‘Can you tell me how you spend your time with your family?’
‘As a father, what do you do with your family?’
3. Working style
‘Can you tell me about your current working style?’
‘What is your role as a doctor, in the workplace or outside the workplace, such as academic societies and medical associations?’
4. Work-Family balance/conflicts/facilitation
‘How do you manage to balance work and family life?’
‘Do you encounter any difficulties or conflicts in balancing your family role with your professional role?’
‘Have you experienced a positive impact at work because of your family role, or a positive impact on your family role because of your professional role?’
5. Becoming a father
‘Before the birth of your child, how much did you foresee yourself being involved in childcare?’
‘How would you describe your experience of childcare?’
‘Has the experience of parenthood changed the image of the father you want to be?’
6. Childcare and work
‘How did your working style change after you became involved in childcare?’
‘Have your views and values about work changed since you became involved in childcare?’
‘Has the experience of being involved in childcare influenced your identity as a doctor?’
7. Message to colleagues
‘Do you have any message for colleagues who are struggling to balance work and family life?’

### Data analysis

We considered that the data collected through the interviews in this study would contain narratives reflecting how the narrators were positioned within existing norms and discourses in a particular society [38]. Using this lens, we analyzed the data in the following four steps: First, after data immersion and familiarization, NK extracted the raw narrative fragments of the research question and assigned each a theme (a form of language that was more abstract than the original data). Second, NK constructed individual storylines based on the identified themes. Third, four other researchers reviewed the themes and storylines presented by NK against the original data to examine their appropriateness. Finally, the researchers cross-read all participants’ storylines and themes and extracted core themes and representative narratives.

### Reflexivity

Of the five members of the research team, FT is an educational sociologist specializing in gender studies (particularly men and masculinity studies), and the rest are doctors specializing in general internal medicine or family medicine. HN is a medical education researcher. TM is a woman, and the other four are men. We recognized that the data obtained from the interviews would be constructed through the interaction between the interviewee and the interviewer. Therefore, when analyzing the interviewees’ words, we also considered the interviewers’ reactions. In particular, when analyzing the polysemic and ambiguous words uttered by the interviewees, we not only interpreted them based on the content of the conversation before and after but also paid attention to how they understood the questions posed by the interviewer and what discourse they presented.

All the five team members had experience in childcare. The first author (NK), a male family physician who led the data collection and analysis, experienced single parenting, which is rare for male doctors in Japan, and struggled greatly with work-life balance. As it was anticipated that his personal experiences would be projected onto data collection and analysis, NK shared his reflections at regular research team meetings and other members commented.

### Ethics

The Research Ethics Committee of the Japanese Primary Care Association approved this study (approval number 2017-003). Participants were provided with a written overview of the study, which was explained to them by the researcher via email or orally. Participants’ consent was obtained in a written form. The researcher informed the participants that the interview might cause unexpected emotions or make them feel uncomfortable, and that they could refuse to answer any questions they did not want to answer or cancel in the middle of the interview. They were also informed that they could withdraw from the study at any time up to three months after the end of the interview.

## Results

Participants’ characteristics are shown in Table 2.

**Table 2:** Participants’ characteristics

Pseudonym	Age	Career Grade	Specialty	Job status		Number of children	Age of youngest child
				Participant	Wife		
Akira	30s	Senior Resident	Family medicine	Full-time	Part-time	1	1
Daisuke	40s	*Qualified	Family medicine	Full-time	Part-time	5	3
Kazuki	30s	*Qualified	Family medicine	Full-time	Stay-at-home	5	1
Kenta	30s	*Qualified	Family medicine	Full-time	Stay-at-home	4	0
Makoto	30s	*Qualified	Surgery	Part-time worker and PhD student	Stay-at-home	2	1
Naoki	30s	*Qualified	Surgery	Part-time worker and PhD student	Stay-at-home	2	1
Ryusuke	30s	*Qualified	General. Int. Med.	Full-time	Part-time	2	6
Takeshi	30s	*Qualified	Surgery	Part-time worker and PhD student	Part-time	1	0
Takuya	40s	*Qualified	Em. Med.	Full-time	Part-time	2	0
Toru	30s	*Qualified	General. Int. Med.	Full-time	Full-time	1	1

*Qualified doctors are those who have completed training in their respective specialty

The magnitude of workload at the workplace varied for each participant, and whether they were in positions to control their working conditions also differed. Daisuke, Kenta, and Ryusuke were busy with clinical, teaching, and management work but were in a higher position in the workplace and able to control their working hours with the help of colleagues, including nurses or doctors. Makoto, Naoki, and Takeshi, who were PhD postgraduates and part-time doctors, had less demanding workload and were better able to devote time to their families. Akira had to spend a lot of time practicing and learning as a resident in a busy workplace, whereas Kazuki often worked long hours in an environment with significant medical needs but limited human resources. Takuya needed to adjust his time at home according to his day- or night-shift work in the emergency department. Toru moved from a busy hospital to a new workplace with less overtime, and could spend more time at home.

Based on the purpose of our study and the research question, we identified three distinctive core themes from the data of nine participants around the father–physician negotiation processes of being a caregiver and a physician. However, in the case of Takeshi, we did not obtain any findings directly relevant to the research question due to a lack of narratives regarding his experiences in negotiating his caregiving role or his thoughts on being a physician. The three core themes are demonstrated in detail below using representative narratives.

### Maintaining gendered assumptions of the medical profession without experiencing conflict

We identified the first theme in the narratives of Naoki, Takuya, and Makoto. They did not experience significant conflicts between caregiving and work. However, their negotiations over caregiving tasks were distinctive in that they relied on gender stereotypes to avoid assuming the caregiving role or limiting the extent to which they would participate in caregiving tasks. Instead, they emphasized assuming the role of a breadwinner as their primary role. Consequently, their negotiations not only reproduced gendered division of labor at home, but also contributed to maintaining the gendered assumptions in the medical profession; that is, physicians were mostly men and exempted from domestic responsibilities, which we comprehensively discuss in the second and third themes. The representative narratives below also reveal the reality of physician fathers having difficulty in becoming aware of and correcting their own gender stereotypes, let alone alter gendered assumptions in the medical professions.

Naoki was among the participants whose negotiations included this process. He recognized that his role as a parent was to earn money for the family. Regarding his caregiving role, he stated his thoughts on why he would not assume that as the primary role.

*NK: How do you feel about your own way of interacting with your child as a father? Are you satisfied with it?*

*Naoki: I read a lot of books on childcare, and I believe the father’s role is not yet necessary for my kids.*

*NK: not yet?*

*Naoki: There are like “Mom, Mom, Mom”…No matter what you do now, they call “Mom!”….so I consider that it’s just fine to play with them only when they turn to me… I would play a rough game, like wrestling.*

To interpret “it’s just fine to play with them only when they turn to me” according to the definition of childcare in this study, we can assume that Naoki was involved in less demanding activities with his children, while leaving the more responsible caregiving tasks to his wife. Moreover, this division of household labor between men and women was justified by gender stereotypes.

This type of gender stereotyping was also evident in Takuya’s narrative. In his view, caregiving was challenging for fathers; this perception was attributed to his belief in inherent differences between men and women.

*Takuya: So, it means I needed to switch my mindset, like assigning roles.*

*NK: It’s a matter of mindset.*

*Takuya: Yeah.*

*NK: It’s about how men become fathers.*

*Takuya: Exactly… If kids cannot speak a certain amount of language, or if you can’t reason with them to some extent, it’s tough for me…However, mothers are probably different. Since the relationship between mothers and children starts when children are a part of mothers’’ body, it is basically so intimate. You know, it’s like fathers aren’t like that.*

The following narratives by Makoto exemplify how physician fathers take advantage of gender stereotypes that consider childcare a women’s role by nature to justify prioritizing being a breadwinner over being an involved caregiver, even in circumstances where their working conditions do not impede them from being the latter. Makoto, who lived with his two children and his stay-at-home wife, was a work-first surgeon who enjoyed performing numerous operations and immersed himself in his work. After a successful career as a specialist, he enrolled in a postgraduate doctoral program and began working part-time as a clinician. This coincided with the birth of the couple’s first child. This simultaneous change in work and family roles altered the meaning he attributed to his profession.

*NK: You got involved in parenting. Did it have any impact on your way or identity as a physician?*

*Makoto: Oh, yes, I think it did. Especially before I had children, I was basically a surgeon, a surgeon who operated a lot. But now that I have children, at least the most important thing is no longer my job. It’s my children.*

The authors initially interpreted this narrative about the shift in “the most important thing” as indicative of a behavioral move toward caregiving rather than working. However, when asked about his own roles, he clarified his focus on the financial stability and his role as a provider, rather than as a caregiver:

*Makoto: I think my way of thinking has changed a lot. When you have a child, what you have to take care of is stability. Of course, financially, it would be better to have stability at least.*

His clarification was consistent with the shift in the meaning of his occupation from something that gave him personal fulfillment to a means of providing for his family.

Regarding caregiving, he explained the extent to which he assumed caregiving responsibilities alongside his wife.

*NK: When it comes to the level of involvement in childcare, from what you have said so far, I got the impression that you were already quite dedicated to it from the beginning. Or did you adjust your role as a caregiver according to the situation ?*

*Makoto: Well, as for the involvement, I wanted to commit myself to parenting. However, in any case, mum is so great. I’m not as good as mum. But yet, I suppose I have a role to play … It’s naturally easier for two people to do it than for one, isn’t it?*

Here, although he participated in childcare, he only assisted his wife, who was the primary caregiver. He rationalized his position as a secondary caregiver by bringing up the stereotypical image of mothers.

The authors further revisit the analysis of the interaction between the shift in valuing “the most important thing” and his identity negotiation. First, this shift mirrored a transformation in his perception of his profession, transitioning from self-fulfillment to breadwinning, which he willingly assumed. Second, he defined the limits of men’s caregiving responsibilities by deploying gender stereotypes. Consequently, his identity negotiation, intertwined with an emphasis on his family’s economic stability, revolved around gender biases that ascribe men a breadwinning role, and associate caregiving with women.

Moreover, being able to provide for a stay-at-home wife and two children on a part-time job, an accomplishment that is often unattainable for other occupations in Japan, showcases the economic privilege of physicians. This facet of social class might have shaped his discourse surrounding the division of household labor.

### Maintaining gendered assumptions of the medical profession while experiencing conflict

We identified a second theme in the narratives of Kenta, Akira, Kazuki, and Toru, who tried to be dedicated caregivers while they were expected to be professionals. Encountering significant conflicts between their professional and childcare responsibilities did not drive them to challenge professional role expectations, which were based on the gendered assumptions that physicians should be exempted from domestic roles. Consequently, they prioritized their professional role over childcare roles, leaving most of the childcare duties to their wives, and ended up being complicit in maintaining the gendered assumptions in the medical professions, as well as the gendered division of labor in the household. We present representative narratives extracted from Kazuki and Akira’s data to illustrate this theme.

Kazuki, a family doctor, lives with his stay-at-home wife and their five children. Despite his wish to dedicate more time to childcare, the demands of his work environment, compounded by a shortage of staff, hindered him from realizing it. During the interview, when asked about the balance between his work and family life, he expressed how he was frustrated by the reality that he had to prioritize his work, leaving much of the childcare to his wife.

*Kazuki: Basically, though, I don’t want to work that much. It’s a contradiction. I feel I have to work, but I want to spend more time with my family than with my work. … But there is more work to be done, and I have no choice but to do it.*

*I know now is the right time for me to get closer to my own children… I feel conflicted about the fact that I can’t find the right balance.*

He then compared the two options of “changing” or ”maintaining” the work-family balance.

*Kazuki: I wonder how much I can do with my children.*

*NK: Do you mean, even if you have time, you wonder how much you can do ?*

*Kazuki: Yeah, just spending an hour with my children in the morning is enough to make me tired. It feels like, my wife is amazing, she spends a lot of time with kids and takes care of them all day long… It’s hard work.*

*Well, the family itself… is a little bit unstable, not balanced, but it’s kind of stable to some extent. For my life, I want to be a bit closer to them. But… I’m worried about the environment, which makes it a bit difficult to limit my working hours.*

Unlike the cases in the first theme, Kazuki experienced conflict between childcare and his professional role. He perceived the state of his family as unbalanced for leaving most of the hard work of childcare—which would leave him exhausted after an hour—to his wife, and would like to be closer to his family. However, due to an overwhelmingly busy work environment, he eventually failed to change his work/family balance, and his negotiations over caregiving and professional roles served to maintain the gendered division of household labor. Moreover, in comparison to the narratives within the first theme, Kazuki’s accounts underscored the dynamics between professional expectations and caregiving responsibilities at home. His unsuccessful attempt to get more involved in caregiving suggests that the medical profession does not assume doctors who have caregiving obligations at home.

Akira, a family medicine resident and father of a one-year-old girl, was busy with training and clinical duties. He wanted to be involved in childcare; therefore, when he returned home from work, he was actively involved with his daughter, feeding her, bathing her, playing with her, and putting her in bed. Meanwhile, it was also important for him to continue his learning in the professional community as a resident. His wife, who worked part-time, expected him to take the child to the nursery in the morning. However, this role created a time conflict with the regular early morning education sessions provided by his workplace.

*Akira: That one hour (the early morning education session) is quite important to me…. It was hard to cut back. So, I talked about it with my wife last time, and she said, ‘Well, it can’t be helped, so I’ll do it (take the kid to nursery). … As such, she kindly says that, so I’m very grateful for that.*

He was exempt from part of his caregiving role, and his wife replaced it in a way that allowed him to focus on the demands of his profession as a doctor. The dynamics of Akira’s narrative match those of Kazuki’s. The profession required doctors to participate in professional activities on the assumption that they would be excused from care roles, such as childcare at home. In other words, we can observe the professional community’s assumption about the timetabling of educational sessions for trainees; trainees are not supposed to take on caregiving roles at home, so they can attend the session even if it is scheduled early in the morning or late in the evening. However, Akira was also expected to take care of the children, which leads to the conflicts described above. To resolve the conflict, the man and his wife choose a solution in line with the gendered division of labor. Consequently, Akira maintained gendered assumptions of professionalism to avoid marginalization by the professional community.

### Deconstructing gendered assumptions of the medical profession through conflict

We identified a third theme in Daisuke and Ryusuke’s narratives. Their negotiations involved questioning and dismantling the assumptions about being a physician and a father when they experienced conflicts between childcare and work. We named this theme the “deconstruction of gendered assumptions.”

While many physician fathers had difficulty undertaking the caregiving role, as discussed in the second theme, Daisuke’s negotiations underwent a significant change. Daisuke remarked that he used to leave home early in the morning, return home late at night, and never felt a problem. His perception, however, changed dramatically when he was placed in a situation in which he had to take care of his children while his wife was hospitalized for a month. During this period, he was responsible for picking up his children from kindergarten by 5 p.m.

*Daisuke: If I had to go home [this early] every day, not just today, I thought that would be a serious situation.*

This experience led him to question workplace customs, which assumed that workers did not play careging roles at home. He then adjusted his hours so that he could devote sufficient time to her care and still fulfill his professional responsibilities between 9:00 am and 5:00 pm.

Ryusuke, a general internal medicine doctor and father of two children, reflectively narrated how he questioned the beliefs that he had held as a doctor and how he reconstructed them.

He said that he used to be a man who devoted everything to his work.

*Ryusuke: Basically, I thought that work was everything. I really enjoyed my work, and I thought that being a doctor meant staying in the hospital all the time, so most of the time I would only go home for a few hours on the weekends, just to sleep.*

For Ryusuke, the image of the ideal doctor was that of one with strict and self-sacrificing work ethics. During the interview, when Ryusuke and the interviewer talked about how doctors were influenced by such professional norms, he also shared his own narrative about the process of learning them.

*Ryusuke: I’ve learned in various places, verbally and non-verbally, that it’s great to stay in the hospital and stay committed to the patients, since I was a junior resident…. they say that doctors who go home early are not good.*

*Looking back, the reason why I wanted to work so hard at the hospital was because it made me very happy to be needed by patients. I felt it very rewarding to support people who would be in danger if I were not there.*

Ryusuke imbibed strong work ethics from the professional community, where value was placed on being close to the patient for as long as possible. He embodied it and worked in situations where his presence or absence affected the health outcomes of his patients, bringing him a sense of self-fulfillment and thus reinforcing the norm. However, the birth of his children made him rethink such a work ethic.

*Ryusuke: There are a lot of things that happen when we have children. At first, they didn’t really listen to me, or it simply was physically hard. I had a lot of conflicts. I felt that my work would be ruined; my professional career, I mean. I had the feeling that it would be weak. … I would go far from the way doctors should be. Doctors are only doctors because they work in a hospital all the time. To be honest, I had looked down on people who were only in the hospital for a short time or left immediately.*

“The way doctors should be,” that Ryusuke had learned and embodied in the professional community, was an altruistic attitude aimed at maximizing clinical outcomes for patients. This was firmly linked to the norm that doctors should serve patients in a self-sacrificing manner, even at the expense of their own family time. In other words, professional norms were implicitly linked to the assumption that doctors must be exempt from caregiving roles at home, justifying the privileges of men in patriarchal family structures. Additionally, his reflection on his previous attitude of disdaining doctors who left work early suggests that doctors’ working styles served as a criterion for evaluating their legitimacy as medical professionals. His attempts to take on a caregiving role broke this assumption, and thus, he could no longer embody professional norms. Here in Ryusuke’s narrative, the profession began to separate itself from the gendered assumption; a new professionalism was reconstructed in which doctors would practice medical care for their patients without the “work in a hospital all the time” norm that had assumed a specific gender role.

*NK: Now, how do you feel about your work style and the way you work for patients ?*

*Ryusuke: Of course, I don’t think at all that it’s okay to do nothing for the patients or compromise the quality of their treatment or care. But it’s not something that I have to deal with on my own, there are nurses and other medical staff, and it’s not just about the time you spend in the hospital. It’s about whether you’re making a difference to the quality of care for patients… We also have to make a good contribution to our families. I have come to believe that it is more important to find a way to help the patient in that limited time.*

## Discussion

This study provides novel and unique knowledge in the academic field of gender and the medical profession by describing the processes of how gender gaps are maintained and by exploring these processes specifically in men’s experience. The findings indicate that physician fathers’ negotiations about being a caregiver often ended up reproducing patriarchal family structures, with the limitation of the extent of men’s involvement in caregiving being leveraged by gender stereotypes and gendered assumptions of the medical profession. Moreover, physicians were often unaware of the extent to which the professionalism they internalized and embodied was inherently gendered, while some acknowledged that they stood upon gendered assumptions but encountered challenges in changing them. In other words, gender gaps in the medical profession continued because being a man exempted from care responsibilities at home is a prerequisite for “the way doctors should be” or medical professionalism.

This argument of male physicians’ exemption from caregiving roles at home aligns with the larger discourse on gender roles and expectations, which often associates femininity with nurturance and domestic roles [10, 39]. Therefore, further studies in this area could benefit from exploring not only the link between masculinity and work ethics but also the complexities of expectations and negotiations surrounding women’s lives. Specifically, our research emphasizes the need to comprehensively examine the specific dynamics of power within relationships involving male doctors and their spouses, as well as the significance of accounting for class-related aspects in domestic roles negotiations within couples.

In addition to this examination into the gender relation within households, our study also provides new insights into power relation within the medical field. The embodiment of professionalism at the individual level was associated with praise for working long hours. This idea, underpinned by the gendered assumptions in the profession, served as a criterion to evaluate the individual doctors’ legitimacy, and contributed to the hierarchization within the medical community based on their working styles and gender. Although hierarchies exist between full-time and part-time workers in other occupations [40, 41], this complex interplay of professionalism, work style, and gender surrounding hierarchization within doctors can be considered unique to the medical profession.

Furthermore, the narratives of the participants illustrated how the workplace norms around time significantly influenced their negotiations. Specifically, our study reveals that the timetabling custom of educational sessions at the workplace was influenced by gendered assumptions, providing another perspective on the link between working time and gender in the medical profession [15].

Having explored the interrelationship between masculinity and the medical profession, our study serves as a foundational step for future research in this area. Specifically, our examination on medical professionalism; the power dynamics between doctors and their spouses, and within the medical profession; class dynamics inherent in the medical field; and the unquestioned norms related to medical education in the workplace, provide a starting point for investigation into the masculinized assumptions unique to the medical profession, compared with other professions.

The narratives in our study also demonstrate the possibility of deconstructing these gendered assumptions at an individual level. In other words, individuals can critically examine and question their beliefs associated with gender roles and expectations, leading to the possibility of reconstructing a new norm to which they choose to conform. Although these findings were identified in the context of Japan, where the gender gap is relatively large, we believe that they have the potential to provide important insights when analyzing the situation in other countries.

### Implications

Based on the findings, we propose three steps for achieving gender equality in the medical profession. First, we need to address the unconscious gender bias of male medical students and male doctors, especially concerning the division of labor at home. As shown in the above results, physician fathers are not entirely free from the classic stereotypes that men work to earn and women take care of children. Although many female doctors worry about the moment when they will have children and the impact of caring roles on their future career trajectories [42], male doctors may have little idea that childcare will affect their career development. These assumptions about gender are usually formed before students enroll in medical school [43], yet we believe that medical teachers can play an important role in addressing unconscious beliefs. Recently, several researchers have proposed transformative learning theory as a guide for implementing educational strategies in relation to the implicit biases or prejudices held by health professionals [44, 45] and argued that creating a disorienting experience by provoking critical reflection on the assumptions could be one of those strategies [44]. As many men and boys are pushed “away from areas of knowledge with which they ought to be in contact” [46], and as a simple lack of knowledge about gender often acts as a hindrance to men with such disorienting experience, we suggest that medical school teachers first help male medical students and male physicians acquire knowledge about the concepts and facts of gender relations, gender gap, patriarchy, and sexism.

Second, medical professionals must be aware of and critically review the implicit assumptions about being a doctor from a gender-sensitive perspective. To deconstruct this gendered normativity, we believe that increasing doctors’ awareness of this issue is crucial. Based on the findings in our study, we propose the following key questions to help medical professionals gain awareness, not only of the assumptions which are linked to gendered division of labor at home, but also of the taken-for-granted customs or rules in their workplace, which are usually perceived as unrelated to gender yet in reality assume only “care-less” men: *What does “the way doctors should be” mean to you? To what extent does having children make it harder for you to continue working in “the way doctors should be”? And would your answer be different if you were a different gender? If so, what gender privilege or gender characteristics does “the way doctors should be” demand you to exercise? Do the customs (such as role allocation within colleagues or the way of scheduling of educational sessions) in your workplace fit both men and women? How does gender interplay with medical professionalism in your workplace and in your country?*

Third, scholars should conduct further research in various contexts. Although we believe that our study included some findings that can be applied to other settings, the realities of the relationship between the medical profession and gender should be explored in different socio-cultural and economic contexts.

### Limitations

One of the limitations of our study is the lack of cases involving male physicians whose wives were employed full-time or who were single parents responsible for raising their children on their own. While this study’s participant group, whose wives were either full-time housewives or part-time workers, certainly reflects the general reality of male physicians in Japan [23], it is necessary to deepen our knowledge of this subject by studying a very small number of cases. Differences in the ratio of men to women in different specialties should also be considered. In surgery, internal medicine, and emergency specialties, the departments to which many of the participants in this study belonged, men comprised more than 80% of the population in Japan [47]. No official data were available on the proportions of men and women in general practice. Further research is required to clarify the link between professional norms and gender norms in different specialties.

Furthermore, an additional limitation of our study is the lack of interviews with the participants’ wives, as well as the limited exploration of participants’ viewpoints regarding their wives’ engagement in domestic tasks, which could have offered alternative perspectives on the realities we explored. Although research regarding middle-class families in Japan has shown that many wives perceive significant disparities in childcare and household burdens as “inevitable” or rational [48], few studies have addressed these mutual expectations between husbands and wives of heterosexual couples in the medical profession. Conducting further research on physicians’ spouses is essential to gain insight into this aspect.

## Conclusions

Exploring the experience of Japanese physician fathers, the study revealed how “the way doctors should be,“ or professional norms, implicitly assume that doctors must be exempt from a caregiving role at home. To promote gender equality, medical students and doctors must question their own unconscious biases regarding gender, and deconstruct gendered assumptions in the medical profession.

## Data Availability

The datasets generated and/or analyzed during the current study are not publicly available as they contain privacy information but are available from the corresponding author on reasonable request.

## References

[1] Pelley E, Carnes M (2020). When a specialty becomes "women’s work": trends in and implications of specialty gender segregation in medicine. Acad Med.

[2] Raj A, Kumra T, Darmstadt GL, Freund KM (2019). Achieving gender and social equality: more than gender parity is needed. Acad Med.

[3] British Medical Association. Sexism in medicine. 2021. https://www.bma.org.uk/advice-and-support/equality-and-diversity-guidance/gender-equality-in-medicine/sexism-in-medicine-report. Accessed 19 Oct 2023.

[4] Hartmann H. Capitalism, patriarchy, and job segregation by sex. Signs. 1;3:137–69.

[5] Frader LL. Gender and labor in world history. In: Meade TA, Wiesner-Hanks ME, editors. A companion to global gender history. 2nd ed. Wiley; 2020. pp. 27–42.

[6] Cruess RL, Cruess SR. Professionalism and professional identity formation: the cognitive base. In: Cruess RL, Cruess SR, Steinert Y, editors. Teaching medical professionalism: supporting the development of a professional Identity. 2nd ed. Cambridge University Press; 2016. p. 5–25.

[7] Riska E. Gender and medical education. In: Brosnan C, Turner BS, editors. Handbook of the sociology of medical education. Routledge; 2009. pp. 89–105.

[8] Hill E, Solomon Y, Dornan T, Stalmeijer R (2015). You become a man in a man’s world’: is there discursive space for women in Surgery?. Med Educ.

[9] Mann KV, Gaufberg E. Role modeling and mentoring in the formation of professional identity. In: Cruess RL, Cruess SR, Steinert Y, editors. Teaching medical professionalism: supporting the development of a professional identity. 2nd ed. Cambridge University Press; 2016. p. 84–96.

[10] Matsui T, Sato M, Kato Y, Nishigori H (2019). Professional identity formation of female doctors in Japan – gap between the married and unmarried. BMC Med Educ.

[11] Volpe RL, Hopkins M, Haidet P, Wolpaw DR, Adams NE (2019). Is research on professional identity formation biased? Early insights from a scoping review and metasynthesis. Med Educ.

[12] Lempp H, Seale C (2004). The hidden curriculum in undergraduate medical education: qualitative study of medical students’ perceptions of teaching. BMJ.

[13] Martimianakis MA, Maniate JM, Hodges BD (2009). Sociological interpretations of professionalism. Med Educ.

[14] De Simone S, Scano C (2018). Discourses of sameness, unbalance and influence: dominant gender order in medicine. Indian J Gend Stud.

[15] Ozbilgin MF, Tsouroufli M, Smith M (2011). Understanding the interplay of time, gender and professionalism in hospital medicine in the UK. Soc Sci Med.

[16] Shelton BA, John D (1996). The Division of Household Labor. Annu Rev Sociol.

[17] Klein M (2016). Educational Expansion, Occupational Closure and the relation between Educational Attainment and Occupational Prestige over Time. Sociology.

[18] U.S. Bureau of Labor Statistics. National occupational employment and wage estimates. 2023. https://www.bls.gov/oes/current/oes_nat.htm. Accessed 19 Oct 2023.

[19] Rosta J, Rø KI (2023). Changes in weekly working hours, proportion of doctors with hours above the limitations of European Working Time Directive (EWTD) and time spent on direct patient care for doctors in Norway from 2016 to 2019: a study based on repeated surveys. BMJ Open.

[20] Statistics Bureau of Japan. 2016 survey on time use and leisure activities: summary of results (Questionnaire A), p.11. https://www.stat.go.jp/english/data/shakai/2016/pdf/timeuse-a2016.pdf. Accessed 7 June 2023.

[21] Gender Equality Bureau. The white paper on gender equality 2023. Cabinet Office, Government of Japan. https://www.gender.go.jp/about_danjo/whitepaper/r05/gaiyou/pdf/r05_gaiyou_en.pdf. Accessed 1 Aug 2023.

[22] Yasukawa K, Nomura K (2012). The division of labor by sex among Japanese physicians. Igaku Kyoiku [Medical Education].

[23] Nakamura M. The determinants of working hours of Japanese female physicians: the effects of family structures and transitions into part-time status. Shakaikagaku Kenkyu [Journal of Social Science]. 2012. https://jww.iss.u-tokyo.ac.jp/jss/64/01/jss6401_045068.html. Accessed 7 June 2023. (Japanese).

[24] Global Gender Gap Report. 2023. World Economic Forum. 2023. https://www.weforum.org/reports/global-gender-gap-report-2023. Accessed 8 July 2023.

[25] Elliott K (2015). Caring masculinities: theorizing an emerging Concept. Men and Masculinities.

[26] Hunter SC, Riggs DW, Augoustinos M (2017). Hegemonic masculinity versus a caring masculinity: implications for understanding primary caregiving fathers. Soc Personal Psychol Compass.

[27] Mori M, Matano H, Mogi T, Mori T, Takeda K, Kaku A et al. “Ikumen-ishi” funtochu [Male physicians are struggling with childcare]. New Medical World Weekly. 5 Aug 2013. https://www.igaku-shoin.co.jp/paper/archive/y2013/PA03038_01. Accessed 7 Jun 2023. (Japanese).

[28] Hasunuma N, Iwama H. Kagayakeru career keisei no kokoroe [Tips on how to build a brilliant career]. New Medical World Weekly. 12 Aug 2019. https://www.igaku-shoin.co.jp/paper/archive/y2019/PA03334_01. Accessed 7 Jun 2023. (Japanese).

[29] Varpio L, Paradis E, Uijtdehaage S, Young M (2020). The distinctions between theory, theoretical framework, and conceptual framework. Acad Med.

[30] West C, Zimmerman DH (1987). Doing gender. Gend Soc.

[31] Connell RW, Messerschmidt JW (2005). Hegemonic masculinity: Rethinking the concept. Gend Soc.

[32] Witz A (1992). Professions and patriarchy.

[33] Sechiyama K. Patriarchy in East Asia: a comparative sociology of gender. BRILL; 2013.

[34] Funahashi K. A comparative sociological examination of contemporary father roles. In: Kuroyanagi H, Yamamoto M, Wakao Y, editors. Fathers and families: questioning fatherhood. Waseda University Press; 1998. pp. 136–68. (Japanese).

[35] Makino K. Kosodate ni huan wo kanjiru oyatachi e [For parents concerned about raising children]. Kyoto: Minerva Shobo; 2005. (Japanese).

[36] OECD Family Database. OECD. 2022. https://www.oecd.org/els/family/SF_2_4_Share_births_outside_marriage.pdf. Accessed 1 Aug 2023.

[37] The Mainichi. Japan’s same-sex couples hope to foster children, but prejudice remains barrier. Mainichi Japan. 27. May 2022. https://mainichi.jp/english/articles/20220526/p2a/00m/0na/022000c. Accessed 1 Aug 2023.

[38] Konopasky A, Varpio L, Stalmeijer RE (2021). The potential of narrative analysis for HPE research: highlighting five analytic lenses. Med Educ.

[39] Long SO, Imamura AE (1996). Nurturing and femininity: The ideal of caregiving in postwar Japan. Re-imaging Japanese women.

[40] Stamper CL, Van Dyne L (2003). Organizational citizenship: a comparison between part-time and full-Time Service employees. Cornell Hotel Restaur Adm Q.

[41] Epstein CF, Seron C, Oglensky B, Sauté R. The part-time paradox: time norms, professional life, family and gender. Routledge; 2014.

[42] Matsui T, Inoue M (2023). Perspectives of medical students on future work-life balance in Japan: a qualitative study using postlecture comments. J Gen Fam Med.

[43] Plan International Japan. Stepping out of the gender box. 2022. https://www.plan-international.jp/news/info/pdf/0415_Gender_stereotype_report_Final.pdf. Accessed 1 Aug 2023. (Japanese)

[44] Sukhera J, Watling CJ, Gonzalez CM (2020). Implicit Bias in Health professions: from Recognition to Transformation. Acad Med.

[45] Soklaridis S, Lin E, Black G, Paton M, LeBlanc C, Besa R (2022). Moving beyond 'think leadership, think white male’: the contents and contexts of equity, diversity and inclusion in physician leadership programmes. BMJ Lead.

[46] Connell RW. The men and the boys. University of California Press; 2000. p. 259.

[47] Ministry of Health, Labour and Welfare Japan. Statistics of physicians, dentists and pharmacists. 2018. https://www.mhlw.go.jp/toukei/saikin/hw/ishi/18/dl/kekka-1.pdf. Accessed 7 June 2023. (Japanese)

[48] Mary C (2022). Brinton. Shibarareru Nihonjin [Japan tied up in Knots].

